# Fecal carriage and multidrug resistance profiles of zoonotic *Campylobacter* species isolated from broilers in Nsukka, Nigeria

**DOI:** 10.14202/vetworld.2025.3409-3419

**Published:** 2025-11-23

**Authors:** Emmanuel O. Njoga, Emmanuel Ochi, Obichukwu C. Nwobi, Joel C. Ugwunwarua, Ebube C. Anidobe, Onyinye S. Onwumere-Idolor, Jameslove I. Kperegbeyi, Everest O. Atadiose, Temitope M. Ogunniran, Ekene V. Ezenduka, James W. Oguttu

**Affiliations:** 1 Department of Agriculture and Animal Health, College of Agriculture and Environmental Sciences, Florida (Science) Campus, University of South Africa, Johannesburg, South Africa; 2 Department of Veterinary Public Health and Preventive Medicine, Faculty of Veterinary Medicine, University of Nigeria, Nsukka, 410001, Enugu State, Nigeria; 3 Department of Animal Production, Faculty of Agriculture, Southern Delta University, P. M. B. 5, Ozoro, Delta State, Nigeria; 4 Department of Animal Science, Faculty of Agriculture, Delta State University Abraka, PMB 001, Delta State, Nigeria; 5 Department of Veterinary Medicine, Faculty of Veterinary Medicine, University of Nigeria, Nsukka, 410001, Enugu State, Nigeria

**Keywords:** antimicrobial resistance, broilers, *Campylobacter coli*, *Campylobacter jejuni*, food safety, multidrug resistance, One Health

## Abstract

**Background and Aim:**

*Campylobacter jejuni* and *Campylobacter coli* are leading causes of bacterial foodborne gastroenteritis worldwide, with poultry serving as a principal reservoir. The rapid emergence of antimicrobial-resistant (AMR) *Campylobacter* strains poses a growing public-health challenge, especially in developing countries where therapeutic options are limited. This study investigated the fecal carriage and AMR profiles of zoonotic *Campylobacter* species (ZCS) isolated from broilers.

**Materials and Methods:**

A cross-sectional study was conducted between February and July 2024. A total of 370 broiler fecal samples were collected using systematic random sampling and cultured on Modified Charcoal Cefoperazone Deoxycholate Agar. Phenotypic identification was performed by colony morphology, Gram staining, and biochemical tests. Antimicrobial susceptibility was assessed using the Kirby–Bauer disk diffusion method against nine antibiotics from distinct classes. Multiple antibiotic resistance (MAR) indices were calculated, and Fisher’s exact test was applied to determine statistical associations (p < 0.05).

**Results:**

*Campylobacter* spp. were detected in 20% (74/370) of samples, comprising *C. jejuni* 6% (22/370) and *C. coli* 14% (52/370). Nearly all isolates (97.3%) exhibited multidrug resistance (MDR), with MAR indices ranging from 0.2 to 1.0 (mean = 0.8). Thirteen distinct AMR patterns were observed; seven were associated with *C. coli* and six with *C. jejuni*. The three most effective antibiotics were gentamicin (GEN) > ciprofloxacin > tetracycline (TET), though *C. coli* isolates were significantly more resistant to GEN (p = 0.001) and TET (p = 0.018).

**Conclusion:**

The 20% fecal carriage of ZCS in slaughtered broilers and the 97.3% MDR prevalence pose a serious public-health and food-safety threat. Prudent antimicrobial use strengthened farm biosecurity, and active AMR surveillance under a One Health framework are urgently needed to curb the spread of antibiotic-resistant *Campylobacter* spp. in poultry production systems and to safeguard human health.

## INTRODUCTION

*Campylobacter* species are Gram-negative, spiral or curved short rods belonging to the family *Campylobacteriaceae* within the class *Epsilonproteobacteria*. They typically measure 0.5–5 μm in length and 0.2–0.8 μm in width [1]. Most zoonotic *Campylobacter* species (ZCS), particularly *Campylobacter jejuni* and *Campylobacter coli*, are thermotolerant and thrive under microaerophilic conditions (5% O_2_, 10% CO_2_, and 85% N_2_) at an optimum temperature of 42°C ± 1°C [1]. The organisms were first described in 1886 by the German–Austrian pediatrician Theodor Escherich, who observed spiral-shaped bacteria in fecal samples from children suffering from gastroenteritis [2, 3]. He referred to the illness as “summer complaint” and named the causative agent *Cholera infantum* [4]. Since then, ZCS, especially *C. jejuni* and *C. coli*, have remained the predominant etiological agents of bacterial foodborne gastroenteritis globally [5], with food-producing animals, most notably poultry, serving as their principal reservoirs [6].

Human infection with ZCS occurs mainly through the fecal–oral route or occupational exposure. In otherwise healthy adults, *Campylobacter* infection commonly manifests as self-limiting gastroenteritis, but in children under 5 years and the elderly, it can lead to severe diarrhea, dysentery, or even death [7]. In animals, transmission occurs primarily via the ingestion of contaminated food or water. Notably, *C. fetus* and *C. jejuni* may also spread through coitus, especially among cattle and small ruminants, contributing to the so-called “community bull syndrome” [8–10]. In livestock, *Campylobacter* infection is associated with reproductive disorders, including late-term abortion, repeat breeding, and infertility [8–10]. A hypervirulent tetracycline (TET)-resistant *C. jejuni* clone, responsible for high abortion rates in sheep in the United States, exemplifies the zoonotic and antimicrobial resistance (AMR) potential of these pathogens [8].

The global and public health significance of ZCS is greatly amplified by their ability to develop resistance to chemotherapeutic agents, particularly fluoroquinolones, macrolides, and TETs, which are frontline drugs against *Campylobacter*iosis [11]. In broilers slaughtered for human consumption, AMR *Campylobacter* strains represent a serious food safety threat, as infections are linked to post-infectious complications such as Guillain–Barré syndrome and reactive arthritis [12–14]. Globally, more than 70% of broilers harbor *C. jejuni* or *C. coli*, and over half of these isolates exhibit resistance to ciprofloxacin (CIP) or erythromycin (ERY), increase the likelihood of treatment failure [15]. Such resistance prolongs illness, elevates morbidity, and inflates healthcare costs [15].

In Nigeria, inadequate biosecurity and hygiene measures in slaughterhouses contribute to the spread of antibiotic-resistant ZCS [16–19]. Poor waste management and the discharge of contaminated effluents can promote environmental dissemination and horizontal gene transfer of AMR determinants among bacterial populations [20]. Genomic analyses of *Campylobacter* isolates from Nigeria have revealed conserved resistance mutations such as *gyrA* (C257T) and *erm(B)*, consistent with global AMR trends [21, 22]. Given the alarming AMR prevalence, *Campylobacter*-related foodborne diseases could escalate into a major public health crisis. Hence, active AMR surveillance in food-producing animals and enhanced antimicrobial stewardship are urgently required to mitigate this threat.

The economic consequences of antimicrobial-resistant *Campylobacter* infections are equally significant. Direct costs stem from prolonged hospital stays, use of second-line antibiotics, and extended outpatient care [23, 24]. Indirect costs include productivity losses due to absenteeism, long-term disability, and premature mortality. Economic models estimate that AMR-associated *Campylobacteriosis* incurs productivity losses exceeding USD 100 million annually, largely due to increased healthcare expenses and diminished workforce efficiency [25, 26].

Despite the recognized role of *C. jejuni* and *C. coli* as major foodborne pathogens and the increasing global concern over AMR in ZCS, data on the prevalence and AMR profiles of *Campylobacter* species from broilers slaughtered for human consumption in Nigeria remain limited and geographically fragmented. Previous Nigerian studies have mostly focused on live birds or retail meats, with little attention to municipal slaughterhouses, which are critical nodes where zoonotic transmission to humans and environmental contamination are most likely to occur. Moreover, most existing investigations have been restricted to phenotypic isolation without a comprehensive evaluation of multiple antibiotic resistance (MAR) indices or comparative species-level resistance patterns. The absence of recent, location-specific data from Nsukka, a major poultry production and marketing hub in southeastern Nigeria, creates a substantial knowledge gap regarding the epidemiology of multidrug-resistant (MDR) *Campylobacter* species in this high-risk environment. In addition, the lack of integrated One Health-based surveillance and systematic antimicrobial stewardship programs in the region impedes early detection and control of AMR dissemination across the animal–human–environment continuum. Addressing this evidence gap is vital for understanding the magnitude of the problem and guiding policy actions aimed at reducing zoonotic transmission and therapeutic failures in both veterinary and human health sectors.

This study aimed to determine the fecal carriage rate and AMR profiles of zoonotic *C. jejuni* and *C. coli* isolated from broilers at the Nsukka Municipal slaughterhouse, Nigeria. Specifically, the research sought to:

1. Estimate the prevalence of *Campylobacter* species in broiler fecal samples collected at slaughter.
2. Characterize the antimicrobial susceptibility profiles of phenotypically identified *C. jejuni* and *C. coli* isolates against commonly used antibiotics in veterinary and human medicine.
3. Determine the MAR indices and identify prevalent resistance patterns among the isolates.
4. Compare resistance trends between *C. jejuni* and *C. coli* to identify species-specific variations in AMR.

The outcomes of this study are expected to provide baseline epidemiological evidence for designing regional AMR surveillance frameworks, strengthening antimicrobial stewardship, and informing One Health interventions aimed at mitigating the public health risks posed by ZCS in Nigeria.

## MATERIALS AND METHODS

### Ethical approval

The study protocol was reviewed and approved by the Research Ethics Committee of the Ministry of Health, Enugu State, Nigeria (Reference No. MH/MSD/REC21/232). The approval covered all procedures involving the collection of biological specimens from food animals intended for human consumption. The research strictly complied with the ethical principles outlined in the National Code of Health Research Ethics, Federal Ministry of Health, Nigeria, and adhered to the international standards for animal use in research as prescribed by the World Organisation for Animal Health/Office International des Epizooties, 2019 and the World Health Organization, 2021.

Because the investigation involved post-slaughter sampling of broiler fecal contents, no live animals were subjected to experimental manipulation, pain, or distress. All samples were collected immediately after evisceration under hygienic conditions by trained personnel using sterile equipment and standard biosafety procedures.

Verbal authorization for sampling and data collection was obtained from the slaughterhouse management and supervising veterinary officers, and all personnel involved were briefed on biosafety, occupational hygiene, and infection-control measures. The research team ensured that all waste materials were disposed of safely following the Ministry of Health biosafety guidelines to prevent environmental contamination.

Human participants were not involved in this study; therefore, no personal identifiers, clinical data, or human specimens were collected. The ethical approval thus covered only the samples of animal origin and associated microbiological analyses.

### Study period and location

The study was conducted between February and July 2024 at Nsukka municipal slaughterhouse located within the Nsukka agricultural zone of Enugu State, Nigeria. The demographics, geographical coordinates, and topography of this area have previously been described [1, 27, 28].

### Study design

A cross-sectional study design was employed to isolate *Campylobacter* species from broiler fecal samples and to determine the antimicrobial susceptibility patterns of phenotypically identified *C. jejuni* and *C. coli* isolates.

### Sample size determination

The minimum required sample size of 236 broilers was estimated using the Raosoft sample size calculator (available at http://www.raosoft.com/samplesize.html) [29]. The calculation was based on a previously reported *Campylobacter* prevalence of 18.9% [6], a 5% margin of error, and an assumed poultry population exceeding 20,000 birds in the study area. To improve statistical robustness, a total of 370 samples were collected.

### Sample collection and transportation

Fecal samples were collected systematically (1 in every 4 broilers) during slaughter between February and July 2024. Following evisceration, cecal contents were obtained using sterile swab sticks pre-moistened with nutrient broth. Samples were placed in sterile containers that contained ice packs so that transportation could occur under cold-chain conditions to the Animal Health Antimicrobial Resistance Surveillance Sentinel Laboratory, Veterinary Teaching Hospital, University of Nigeria, Nsukka. Samples were analyzed within 3 h of collection.

### Isolation and identification of *Campylobacter* species

Isolation of *Campylobacter* spp. was carried out using Modified Charcoal Cefoperazone Deoxycholate Agar (mCCDA, CMO739, Oxoid, UK) as described by Njoga *et al*. [1]. The medium was prepared according to the manufacturer’s instructions and supplemented with *Campylobacter* selective supplement (SRO155E, Oxoid). Swab samples were streaked directly onto the agar and incubated under microaerophilic conditions generated using CampyGen (CN0025A, Oxoid) at 42°C for 48 h.

Typical colonies were identified based on morphology, flat, thinly spreading, and creamy colonies for *C. jejuni* and glossy and pinpoint colonies for *C. coli*. The identified colonies were subcultured onto fresh mCCDA plates for purification and then subjected to biochemical characterization as described by Barrett *et al*. [30]. Gram-negative, slender, spiral, or comma-shaped rods observed under oil-immersion microscopy were presumptively identified as *Campylobacter* species. Identification was limited to phenotypic and biochemical methods. Molecular confirmation was not performed.

### Antimicrobial susceptibility testing

Antimicrobial susceptibility was determined using the Kirby–Bauer disk diffusion method, as described by Shakir *et al*. [31]. Nine antimicrobial agents representing different antibiotic classes were tested: ERY (15 μg), gentamicin (GEN) (10 μg), penicillin-G (PEN) (10 μg), ceftriaxone (CFX) (30 μg), metronidazole (MTZ) (50 μg), chloramphenicol (CRL) (30 μg), clindamycin (CND) (2 μg), CIP (5 μg), and TET (30 μg). All antibiotic disks were obtained from Oxoid.

Each bacterial suspension was adjusted to 0.5 McFarland standard and evenly spread onto Mueller–Hinton agar (CM0337, Oxoid) plates. The disks were placed on the inoculated surfaces and allowed to diffuse for 30 minutes before incubation under microaerophilic conditions at 37°C for 24 h.

The inhibition zone diameters were measured (mm) and interpreted following the Clinical and Laboratory Standards Institute (CLSI) guidelines, 31**^st^** edition (2021) [32]. Isolates were categorized as susceptible (S), intermediate (I), or resistant (R). For analytical rigor, “intermediate” isolates were reclassified as “resistant,” as recommended by CLSI, due to the higher risk of treatment failure in immunocompromised individuals or infections at sites with limited drug penetration [32].

### Determination of MAR index and MDR

The MAR index was calculated using the formula below [30]:

MAR = R/E

Where R represents the number of antibiotics to which an isolate was resistant, and E denotes the total number of antibiotics tested.

A MAR index >0.2 indicates exposure to high-risk environments with intensive antibiotic usage. Isolates exhibiting resistance to at least one antibiotic in three or more antimicrobial classes were classified as MDR, as described by Magiorakos *et al*. [33].

### Statistical analysis

All data were analyzed using GraphPad Prism version 6.04 (GraphPad Software Inc., San Diego, CA, USA). Fisher’s exact test was applied to determine statistical associations (p < 0.05) between antibiotic susceptibility patterns and individual antimicrobials. Comparative resistance profiles between *C. jejuni* and *C. coli* were similarly evaluated using the same test at a 5% significance level.

## RESULTS

### Prevalence of *Campylobacter* species

**Figure 1:**
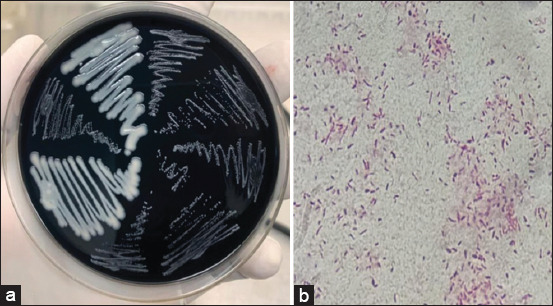
(a) Petri dishes showing thinly spreading swampy colonies appearing creamy/milkish and pin-point colonies appearing glassy on modified Charcoal Cefoperazone Deoxycholate Agar, suggestive of *Campylobacter jejuni* and *Campylobacter coli*, respectively. (b) Gram-negative slender and curved short rods characteristic of *Campylobacter* species under light microscopy at the oil-immersion objective.

Out of the 370 broiler fecal samples analyzed, *Campylobacter* species were detected in 20% (74/370) of the samples. The species-specific prevalence rates were 6% (22/370) for *C. jejuni* and 14% (52/370) for *C. coli*. The colonial morphology and microscopic characteristics typical of *Campylobacter* species observed during the study are presented in Figure 1, showing flat, thinly spreading, creamy colonies (*C. jejuni*) and glossy, pinpoint colonies (*C. coli*), alongside curved, slender rods on Gram staining.

### AMR profile (antibiogram)

Nearly all the *Campylobacter* isolates (97.3%) exhibited MDR, being non-susceptible to at least one antimicrobial agent in three or more antibiotic classes. The overall antimicrobial susceptibility results are presented in Table 1. Among the antibiotics tested, the three agents showing the highest susceptibility rates were GEN > CIP > TET.

**Table 1:** Overall AMR profile of 74 *Campylobacter* species isolated from broilers slaughtered at Nsukka slaughterhouse, Nigeria.

Antimicrobial class	Antimicrobial agents (mg)	Number of resistance (%)	Number of susceptible individuals (%)	p-value
Cephalosporin	CFX (30)	72 (97.3)	2 (2.7)	<0.001*
Beta-lactam	PEN (10)	72 (97.3)	2 (2.7)	
Imidazole	MTZ (50)	72 (97.3)	2 (2.7)	
Macrolide	ERY (15)	56 (75.7)	18 (24.3)	
Aminoglycoside	GEN (10)	38 (51.4)	36 (48.6)	
Chloramphenicol	CRL (30)	56 (75.7)	18 (24.3)	
Lincomycin	CND (2)	58 (78.4)	16 (21.6)	
Fluoroquinolone	CIP (5)	44 (59.5)	30 (40.5)	
Tetracycline	TET (30)	50 (67.6)	24 (32.4)	

* Significant p*-*value; Fisher’s exact test (GraphPad Prism, version 8.0.4, San Diego, CA, USA). CFX = Ceftriaxone, CRL = Chloramphenicol, CIP = Ciprofloxacin, CND = Clindamycin, ERY = Erythromycin, GEN = Gentamicin, MTZ = Metronidazole, PEN = Penicillin G, TET = Tetracycline, AMR = Antimicrobial resistance.

The proportion of isolates resistant to ERY and CRL was equal (75.7% each), while resistance to CND was slightly higher (78.4%). A statistically significant association (p < 0.05) was observed between antimicrobial susceptibility and antibiotic type, confirming significant resistance trends across isolates (Table 1).

Species-level comparison revealed that *C. coli* isolates were significantly more resistant to GEN (p = 0.001) and TET (p = 0.018) than *C. jejuni* isolates (Table 2). Furthermore, all *C. coli* isolates (100%) were resistant to CFX and PEN. These findings highlight the widespread and concerning resistance of zoonotic *Campylobacter* isolates to commonly used antibiotics.

**Table 2:** Comparison of the AMR profiles of 22 *C. jejuni* and 52 *C. coli* isolates from broilers slaughtered for human consumption at Nsukka slaughterhouse, Nigeria.

Antimicrobial agents (mg)	*C. jejuni*		*C. coli*		p-value
	
	Susceptible (%)	Resistant (%)	Susceptible (%)	Resistant (%)	
CFX (30)	0 (0)	22 (100)	2 (3.8)	50 (96.2)	0.998
PEN (10)	0 (0)	22 (100)	2 (3.8)	50 (96.2)	0.998
MTZ (50)	2 (9.1)	20 (90.9)	0 (0)	52 (100)	0.297
ERY (15)	4 (18.2)	18 (81.8)	14 (26.9)	38 (73.1)	0.695
GEN (10)	20 (90.9)	2 (9.1)	16 (30.8)	36 (69.2)	0.001*
CRL (30)	2 (9.1)	20 (90.9)	16 (30.8)	36 (69.2)	0.229
CND (2)	2 (9.1)	20 (90.9)	14 (26.9)	38 (73.1)	0.391
CIP (5)	18 (81.8)	4 (18.2)	14 (26.9)	38 (73.1)	0.695
TET (30)	14 (63.6)	8 (36.4)	10 (19.2)	42 (80.8)	0.018*

* Significant p-value; Fisher’s exact test (GraphPad Prism, version 8.0.4, San Diego, CA, USA). CFX = Ceftriaxone, CRL = Chloramphenicol, CIP = Ciprofloxacin, CND = Clindamycin, ERY = Erythromycin, GEN = Gentamicin, MTZ = Metronidazole, PEN = Penicillin G, TET = Tetracycline, *C. jejuni* = *Campylobacter jejuni*, *C. coli* = *Campylobacter coli*, AMR = Antimicrobial resistance, CI = Confidence interval.

### MAR indices

**Figure 2:**
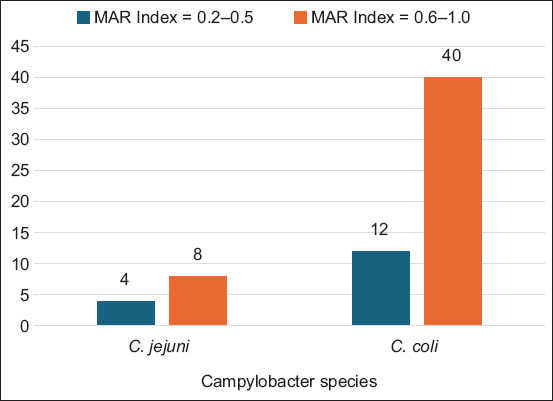
Comparison of the multiple antibiotic resistance indices of *Campylobacter jejuni* (n = 22) and *Campylobacter coli* (n = 52) isolated from broilers slaughtered for human consumption at Nsukka slaughterhouse, Nigeria.

The distribution of MAR indices among the 74 *Campylobacter* isolates is shown in Figure 2. The MAR index values ranged from 0.2 to 1.0, with a mean value of 0.8, indicating a high level of antibiotic exposure and resistance pressure.

For *C. jejuni* isolates, MAR indices ranged from 0.3 to 1.0 (mean = 0.7), whereas *C. coli* isolates ranged from 0.3 to 1.0 (mean = 0.8). A large proportion of isolates exhibited MAR values between 0.6 and 1.0, accounting for 81.8% of *C. jejuni* and 76.9% of *C. coli* isolates. These results suggest that most *Campylobacter* strains originated from high-risk environments with extensive antibiotic usage.

### AMR patterns

Thirteen distinct AMR patterns were identified among the 74 *Campylobacter* isolates (Table 3). The most frequent composite resistance patterns observed were CFX–CRL–CIP–CND–ERY–GEN–MTZ–PEN–TET (18 isolates, 24.3%) and CFX–CRL–CIP–CND–ERY–GEN–MTZ–PEN (10 isolates, 13.5%). The least common pattern was MTZ–PEN, exhibited by only two isolates (2.7%).

**Table 3:** AMR pattern of all 74 *Campylobacter* species isolates from broilers slaughtered for human consumption in Nsukka slaughterhouses, Nigeria.

No.	Phenotypic AMR pattern exhibited	Frequency (%)
1.	MTZ-PEN	2 (2.7)
2.	CFX-MTZ-PEN	8 (10.8)
3.	CFX-MTZ-TET	2 (2.7)
4.	CFX-CND-PEN	2 (2.7)
5.	CFX-CIP-MTZ-PEN	2 (2.7)
6.	CFX-CRL-CND-ERY-MTZ-PEN	8 (10.8)
7.	CFX-CRL-CND-MTZ-PEN-TET	2 (2.7)
8.	CFX-CIP-ERY-MTZ-PEN-TET	2 (2.7)
9.	CFX-CRL-CND-ERY-MTZ-PEN-TET	6 (8.1)
10.	CFX-CRL-CIP-CND-ERY-MTZ-PEN	2 (2.7)
11.	CFX-CRL-CIP-CND-ERY-GEN-MTZ	10 (13.5)
12.	CFX-CRL-CIP-CND-ERY-GEN-MTZ-PET	10 (13.5)
13.	CFX-CRL-CIP-CND-ERY-GEN-MTZ-PEN-TET	18 (24.3)

CFX = Ceftriaxone, CRL = Chloramphenicol, CIP = Ciprofloxacin, CND = Clindamycin, ERY = Erythromycin, GEN = Gentamicin, MTZ = Metronidazole, PEN = Penicillin G, TET = Tetracycline, AMR = Antimicrobial resistance.

Species-specific AMR distributions are summarized in Table 4. Among *C. jejuni* isolates, the most frequent resistance pattern was CFX–CRL–CND–ERY–MTZ–PEN (8 isolates, 36.3%), whereas the longest and most extensive pattern, CFX–CRL–CIP–CND–ERY–GEN–MTZ–PEN–TET, was observed in two isolates.

**Table 4:** AMR pattern exhibited by 22 *C. jejuni* and 52 *C. coli* isolates from broilers slaughtered for human consumption at Nsukka slaughterhouses, Nigeria.

No.	Phenotypic AMR pattern exhibited	Frequency (%)
*C. jejuni* (n = 22)		
1.	CFX-CND-PEN	2 (9.1)
2.	CFX-MTZ-PEN	2 (9.1
3.	CFX-CRL-CND-ERY-MTZ-PEN	8 (36.3)
4.	CFX-CRL-CND-ERY-MTZ-PEN-TET	6 (27.3)
5.	CFX-CRL-CIP-CND-ERY-MTZ-PEN	2 (9.1)
6.	CFX-CRL-CIP-CND-ERY-GEN-MTZ-PEN-TET	2 (9.1)
*C. coli* (n = 52)		
1.	MTZ-PEN	1 (1.9)
2.	CFX-MTX-PEN	5 (9.6)
3.	CFX-MET-TET	11 (21.2)
4.	CFX-CIP-MTZ-PEN	5 (9.6)
5.	CFX-CRL-CND-MTZ-PEN-TET	6 (11.5
6.	CFX-CIP-ERY-MTZ-PEN-TET	10 (19.2)
7.	CFX-CRL-CIP-CND-ERY-GEN-MTZ-PEN-TET	14 (26.9)

CFX = Ceftriaxone, CRL = Chloramphenicol, CIP = Ciprofloxacin, CND = Clindamycin, ERY = Erythromycin, GEN = Gentamicin, MTZ = Metronidazole, PEN = Penicillin G, TET = Tetracycline, *C. jejuni* = *Campylobacter jejuni*, *C. coli* = *Campylobacter coli*, AMR = Antimicrobial resistance.

In *C. coli*, the predominant resistance profiles were CFX–CIP–ERY–MTZ–PEN–TET (10 isolates, 19.2%) and CFX–CRL–CIP–CND–ERY–GEN–MTZ–PEN–TET (18 isolates, 26.9%). Of the 13 identified AMR patterns overall, 7 were associated with *C. coli* and 6 with *C. jejuni*, underscoring species-specific variations in resistance phenotypes.

## DISCUSSION

### Prevalence of *Campylobacter* species and public health significance

The detection of ZCS in 20% of broilers slaughtered for human consumption at the Nsukka municipal slaughterhouse is epidemiologically significant. This translates to one in every five broilers harboring *Campylobacter*, highlighting a major public health and food safety concern. Given the zoonotic potential of *C. jejuni* and *C. coli*, their ease of transmission via the food chain or occupational exposure, and the role of poultry as a key reservoir, this prevalence indicates a substantial risk of human infection in the study area [34].

Human–animal cohabitation, typical of subsistence poultry farming systems in Nigeria, may further facilitate transmission. Broilers are often kept within residential premises by smallholder farmers, increasing exposure risk [35]. In addition, poor occupational hygiene practices, such as the absence of personal protective equipment among slaughterhouse workers, may enhance zoonotic transmission during carcass processing [28, 36, 37].

The *C. jejuni* and *C. coli* strains isolated in this study are responsible for up to 95% of global human *Campylobacteriosis* cases, making them the leading causes of bacterial foodborne gastroenteritis [38–40]. Although *Campylobacteriosis* is often self-limiting in healthy adults, infections can result in severe post-infectious complications such as Guillain–Barré syndrome, Miller–Fischer syndrome, reactive arthritis, Reiter’s syndrome, and inflammatory bowel diseases, including Crohn’s disease and ulcerative colitis [14, 41, 42].

### Food safety implications

From a food safety standpoint, the observed 20% prevalence is alarming, as contamination during slaughter and processing can introduce *Campylobacter* into the human food chain. However, the traditional practice of thoroughly cooking meat in African households, through boiling, frying, or grilling at 80°C–90°C for ≥30 min, serves as a mitigating factor by effectively destroying most bacterial pathogens [43]. This cultural cooking habit likely contributes to the lower frequency of foodborne *Campylobacteriosis* outbreaks in many African communities.

Beyond human health, poultry plays a dual role as both a reservoir and a victim of *Campylobacter* infections. A novel species, *C. hepaticus*, has been reported by Groves *et al*. [44] to cause spotty liver disease in layer hens, resulting in up to 15% mortality and a 35% reduction in egg production. Furthermore, when broilers are reared in proximity to small ruminants or pets, a common rural practice, cross-species transmission can occur. In livestock, *C. jejuni* infection can induce late-term abortions and infertility, leading to significant reproductive losses and posing further zoonotic risks [9, 45].

### Comparative prevalence with other studies

The overall 20% prevalence reported in this study is lower than the 36% previously documented by Akwuoba *et al*. [46] in the same geographical area. This reduction may be attributed to the widespread adoption of intensive poultry production systems, which limit birds’ exposure to high environmental temperatures favorable for *Campylobacter* proliferation [6] and restrict contact with wild or migratory birds that serve as natural reservoirs [6].

The use of prophylactic antimicrobials in poultry farming across Nigeria [47, 48] might also contribute to the reduced detection rate by suppressing subclinical infections. Although prophylactic use can minimize clinical disease, it may inadvertently influence the microbial ecology of the gut, temporarily lower detectable *Campylobacter* levels while promoting AMR.

At the national level, the prevalence aligns with the 22% reported in Ogun State [49] but exceeds the 5.3% recorded in Lagos State [50] and remains lower than the 40% found in Plateau State [51]. Globally, it is also below the rates reported in Peru (36.2%) [52], Ghana (43.1%) [53], United Kingdom (79.2%) [54], Ethiopia (70%) [55], and Iran (25.1%) [56]. These variations may be from differences in diagnostic approaches, biosecurity standards, climatic factors, and researcher expertise, all of which influence *Campylobacter* isolation efficiency.

### AMR patterns

The detection of 97.3% MDR among *Campylobacter* isolates represents a serious public health threat. The extensive resistance to CFX, PEN, and MTZ, drugs often used empirically for gastroenteritis, further underscores the growing ineffectiveness of common therapeutic options. The identification of 13 distinct AMR patterns (7 in *C. coli* and 6 in *C. jejuni*) reflects the adaptive capacity of these pathogens under antibiotic selection pressure.

The high MAR indices (0.2–1.0; mean = 0.8) suggest that most isolates originated from environments with frequent and unregulated antibiotic exposure, consistent with earlier reports linking MAR > 0.2 to high-risk contamination sources [57]. This degree of resistance not only limits therapeutic choices for human infections but also increases the likelihood of treatment failure, chronic infection, and post-infectious complications, particularly among immunocompromised individuals.

The AMR patterns observed in this study mirror those reported by Okunlade *et al*. [58] in Oyo State, Nigeria, where all *Campylobacter* isolates exhibited resistance to amoxicillin, CFX, and ERY. The resistance rates of 59.5% to CIP (fluoroquinolone) and 75.7% to ERY (macrolide) are particularly concerning since these agents remain the first-line treatments for human *Campylobacteriosis* [59]. Their overuse and unregulated availability as over-the-counter “Reserve Group” antibiotics in Nigeria [46, 60] likely contribute to this alarming resistance pattern.

### Drivers and One Health implications of AMR

The emergence and spread of AMR in developing regions are fueled by irrational prescription practices, lack of diagnostic facilities, inadequate public awareness, and weak drug regulation systems [46]. In addition, globalization and cross-border animal trade facilitate the international dissemination of resistant strains [5]. Therefore, implementing a One Health-based strategy, that integrates human, animal, and environmental health, is essential for tackling the AMR crisis in Africa and beyond [5].

The economic burden of AMR *Campylobacter* infections is equally significant. Resistant infections lead to prolonged hospitalization, higher treatment costs, and the need for second-line antibiotics, which are often more expensive and less accessible [61, 62]. In livestock, the consequences extend to reproductive failures, abortion, and reduced productivity, amplifying economic losses and threatening food security [9, 10].

## CONCLUSION

This study provides the first comprehensive evidence on the fecal carriage and AMR profiles of zoonotic *C. jejuni* and *C. coli* isolated from broilers slaughtered for human consumption in the Nsukka municipal slaughterhouse, Nigeria. The overall prevalence of *Campylobacter* species was 20%, comprising 6% *C. jejuni* and 14% *C. coli*, indicating a substantial public health and food safety concern in the region. Nearly all isolates (97.3%) exhibited MDR with a mean MAR index of 0.8, reflecting widespread exposure to antimicrobial agents. High resistance levels were observed against β-lactams, cephalosporins, macrolides, and fluoroquinolones, antibiotic classes critically important to both human and veterinary medicine. The pronounced resistance of *C. coli* to GEN and TET further highlights the adaptive potential of ZCS under sustained antibiotic pressure.

These findings emphasize an urgent need to reinforce antimicrobial stewardship, prudent antibiotic use, and biosecurity measures within poultry farms and slaughterhouses. Institutionalized AMR surveillance, regulated antibiotic distribution, and hygienic slaughter practices can significantly reduce the risk of zoonotic transmission. Improved awareness among farmers, veterinarians, and meat handlers is essential to curtail indiscriminate antibiotic use and enhance food safety.

A key strength of this research lies in its robust sampling design, standardized phenotypic identification, and comprehensive evaluation of species-specific AMR patterns, providing a valuable baseline for regional surveillance and policy formulation. However, the study’s reliance on phenotypic methods without molecular confirmation or resistance gene characterization represents a limitation, and its cross-sectional nature prevents assessment of temporal trends or causal relationships.

Future investigations should integrate molecular typing and whole-genome sequencing to elucidate genetic determinants of resistance and trace transmission dynamics across animal, human, and environmental interfaces. Longitudinal and multi-sectorial surveillance is needed to evaluate the impact of interventions over time.

Summarily, broilers slaughtered for human consumption in Nsukka represent a significant reservoir of MDR *Campylobacter* species. These results reinforce the importance of adopting a One Health approach that links veterinary, environmental, and human health sectors to mitigate AMR, safeguard food safety, and preserve the efficacy of vital antibiotics for future generations.

## DATA AVAILABILITY

All data generated during the study are included in the article.

## AUTHORS’ CONTRIBUTIONS

EON, EO, JCU, OCN, OSO, ECA, and JIK: Methodology and conceptualization. EON and EO: Formal analysis and writing – original draft preparation. EON, EO, JCU, OCN, JCU, EOA, TMO, JWO, and EVE: Investigation. EON, EO, JCU, OCN, JCU, OSO, ECA, JIK, EOA, TMO, EVE, and JWO: Writing – review and editing. EON, OCN, EVE, and JWO: Supervised the study and edited the manuscript. All authors have read and approved the final version of the manuscript.
